# Polyphenolic Components and Phytochemicals Deployed as Nutraceuticals and Pharmafoods

**DOI:** 10.1002/fsn3.72105

**Published:** 2026-07-12

**Authors:** Subhash Chandra, Md. Sakib Al Hasan, Md. A. K. Azad, Atasi Goswami

**Affiliations:** ^1^ Department of Chemistry, School of Basic & Applied Sciences Shri Guru Ram Rai University Dehradun Uttarakhand India; ^2^ Department of Pharmacy Gopalganj Science and Technology University Gopalganj Bangladesh; ^3^ Department of Pharmacy Daffodil International University (DIU) Dhaka Bangladesh; ^4^ Chemistry Discipline Khulna University Khulna Bangladesh

**Keywords:** nutraceuticals, phytochemicals, phytoestrogens, polyphenols, probiotic

## Abstract

Polyphenols have the potential to prevent many diseases physiologically. This review is a summary of the significance and uses of polyphenols in medicine, pharmaceuticals, nutraceuticals, and dietary supplements. We conducted a systematic review of the literature between 2010 and 2025 based on literature searches online and offline. The Cochrane Risk of Bias tool (RoB 2.0) was used to assess the risk of bias in randomized controlled trials. Consequently, polyphenols (berberine, EGCG (green tea), curcumin, luteolin, lycopene, apigenin, quercetin) are used to treat acute and chronic diseases. Polyphenols have been shown to have anti‐cancer, anti‐diabetic, anti‐inflammatory, anti‐analgesic, anti‐bacterial, and immunomodulatory properties, making them potential adjuvants for a variety of treatments. A discussion of nanoparticles' pharmacological applications, clinical evidence, safety, bioavailability, toxicity, and prospects will also be included.

## Graphical abstract

This review (2010–2025) highlights the therapeutic potential of dietary polyphenols against cancer, diabetes, cardiovascular, neurological, and inflammatory diseases. It summarizes molecular mechanisms, clinical evidence, nanoparticle‐based delivery, bioavailability, safety, toxicity, and future prospects supporting polyphenols as promising therapeutic and preventive agents.

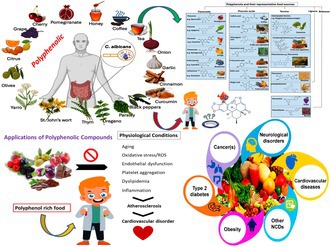

## 1 Introduction

Polyphenols are a large family of naturally occurring phenols and have received a lot of interest since scientists have slowly learned the health benefits of the nutritional content of plants. Tea, coffee, grains, fruits and vegetables are some naturally occurring sources of polyphenols. These chemicals are a general category of flavonoids, total phenolic compounds, tannins, and anthocyanins, which have many different complex structures and uses, and which are based on a phenolic ring as the basic monomer (Zhang et al. 2021; Guo et al. 2022). Different bioactive compounds present in certain foods have the potential to act on the body and have different biological effects. Plants‐based diet contains polyphenols and other bioactive compounds (Hosseini et al. 2022; Zhang et al. 2022). Due to their broad and distinct chemical structure, polyphenols are potential chemical agents for sub‐acute as well as long‐term conditions, as they can have a very broad range of biological effects (Ma and Chen 2020; Ashwin et al. 2021). Polyphenols, which are widely available antioxidants, have been shown to stop the atherosclerosis and slow down the aging process (Fan and Beta 2017). The polyphenol‐rich foods can be used for a wide range of diseases (Mehmood et al. 2020; Garcia‐Conesa and Larrosa 2020) including diabetes, cancer, osteoporosis, neurological diseases, and heart diseases (Ghiringhell et al. 2012; Qiu et al. 2019). They can also be prebiotics with several beneficial impacts on the health of the intestines (Fan et al. 2017; Thilakarathna et al. 2018). They are commonly used in meals that have a functional purpose, which can lead to beneficial outcomes via food. Proteins are a versatile type of bio‐based polymers with a number of desirable properties including nutritional value, foaming, emulsifying, hydrophilic and hydrophobic properties, which can be engineered into nanofibers, nanogels, nanoparticles, nanofilms and nanoemulsions (Guan et al. 2022). Lipid based nanocarriers also have an excellent biocompatibility and biodegradability.

The current context of scientific literature, this review attempts to provide researchers, academics, medical professionals, and pharmaceutical scientists a comprehensive resource to understand the current landscape and future prospects of polyphenols and phytoconstituents for health and medicine. As a result of the oxidative stress‐related diseases, chronic diseases, and antibiotic resistance, a search for reliable and safe natural bioactive compounds has gained momentum. A lot of preclinical and clinical studies show that polyphenols and other phytoconstituents have a great therapeutic potential.

This investigation aims to give a general overview of the relevance and applications of polyphenols in pharmaceuticals, nutraceuticals, dietary supplements and healthcare.

## 2 Sources and Methodology

The literature data was retrieved with Core Collection, Web of Science, Google Scholar, Scopus, Science Direct, PubMed, MDPI, Clarivate Analytical, Google Academic and Scientific Electronic Library Online (SciELO) from 2010 to 2025. The words searched were: “polyphenols”, “pharmafood”, “nutraceuticals”, “probiotics”, “nanoparticle”, and “pharmacological applications”. The published articles retrieved were considered on the basis of their clinical evidence as part of pharmacological applications to promote polyphenols and phytoconstituents. The articles was classified into those reporting (i) the in vitro/in vivo study was an important contribution to society and (ii) validated methods were used in the published study. The publications were only included if they were peer‐reviewed and published in a high‐impact journal that is indexed in Scopus, Web of Science, ScienceDirect and Research Gate. The articles identified and excluded were non‐peer‐reviewed, such as conference abstracts, editorials, undergraduate theses, book chapters and opinion pieces.

## 3 Polyphenols

Polyphenols are the biggest group of secondary metabolites produced by plants through their polyketide or substrate/phenylpropanoid pathway, comprising more than 8000 structural variations (Manach et al. 2004; Sharma et al. 2025). They have beneficial properties to help plants combat pathogens, parasites, and herbivores, and enhance the physiological qualities of fruits, berries, or leaves, as well as products like ultra‐virgin olive oil or alcohol. They can help plants tolerate UV radiation, cold weather and droughts. Natural polyphenols such as phenolic acid and the highly polymerized polyphenols, like condensed tannins (Mitjavila and Moreno 2012) exemplify the structural diversity of polyphenols. The number of phenolic groups and the structural elements that join them determine the main subgroups of polyphenols such as flavonoids, phenolic acids, and the rare lignans and stilbenes (Harborne and Williams 2000). In terms of their relatively simpler structures, the two subclasses of phenolic acids are the cinnamic acids (caffeic and ferulic acids) and hydroxybenzoic acids (gallic, salicylic and vanillic acids). The most diverse of polyphenols are flavonoids. They have a similar structure to diphenylpropanes (C‐6, C‐3 and C‐6), oxygenated heterocycles that consist of three carbons attached to aromatic rings. This polyphenol cosmos is very complicated since it reacts with alcohol, acids and various carbohydrates. Most of the flavonoids are physiologically active substances which are usually found in alcoholic beverages, sugars or foods with acid (Fraga et al. 2010). High prevalence diseases such as cancer and cardiovascular disease are linked with a diet rich in plant foods that has a high low‐grade inflammatory burden (Pant et al. 2025; Goel et al. 2008). Notably, the bioflavors which influence these pathways that can cause low‐grade inflammation have been associated with the beneficial biological effects of grains, legumes, fruits, vegetables, tea and wine (Lewandowska et al. 2016; Calvanese et al. 2009; Tsang and Kwok 2010). A recent study called Prevencion con Dieta Mediterranea (PREDIMED), related to high polyphenol diet intake, has shown that it was associated with reduced mortality, decreased cardiovascular disease and decreased diabetes incidence (Shankar et al. 2016; George et al. 2017).

Interestingly, improved redox state homeostasis (Storniolo et al. 2014) has been associated with the beneficial effects of the Mediterranean diet, and with decreased low‐grade inflammation (Rahman et al. 2006). The polyphenols intake has also been associated with a reduced cancer risk, as shown by epidemiological and clinical studies (Martínez and Moreno 2000). There has been a considerable amount of information on the biological actions of polyphenols, but none that indicates that the actions that they have on the human body are harmful. Until now, only three foods (extra virgin olive oil, chocolate and olives) have a health claim regarding their polyphenols content (Sacanella et al. 2007; Wagner 2006).

Current research suggests that it's the combination of phytochemicals that impart health benefits, not any specific polyphenols. The recent studies on health effects of polyphenols will be discussed. An overview of relevant facts, such as the wide variety of polyphenols structures, their bioactive characteristics, and the challenge of figuring out the amount of polyphenols in food, will be given in the first section. The second part will be dedicated to the bioavailability of polyphenols and polyphenols interactions with other food components, depending on the gut microbiota, culinary techniques and the food composition. Finally, employing molecular pharmacology it will explore the pharmacology of polyphenols and their multiple targets.

### 3.1 Nutraceuticals

**FIGURE 1:**
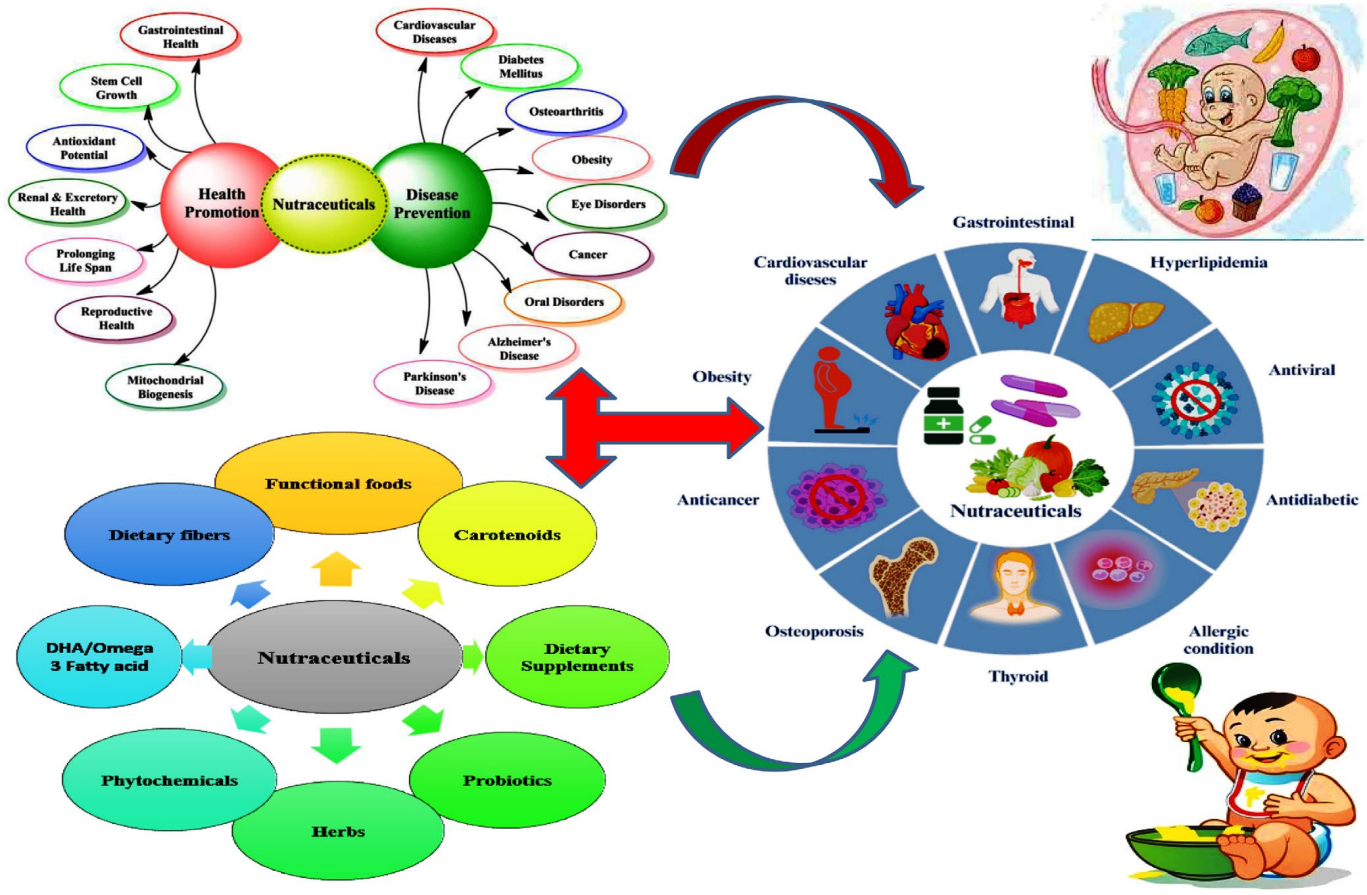
Nutraceutical foods and their applications.

The tenets “nutrition” and “pharmaceutical” are the origins of the expression nutritraceuticals, which was created by Dr. Stephen de Felice and focuses on dietary products and foods that serve as medical and health benefits, such as healthcare and illness prevention (Biesalski 2002). It has been known that nutraceuticals confer protection against chronic disease or have physiological benefits. Phytochemicals are bioactive components found at the intersection of food and medicine that help to maintain and improve health. Examples of these products include items that are made with herbs, processed foods and beverages, separated nutrients, nutritional supplements, genetically engineered designer foods, and special diets (Kalra 2003; Elvira‐Torales et al. 2019). They are important to sustaining the ideal immunological response, and so over‐ or under‐consumption can be harmful to health. Another exciting area of nutraceutical therapy is nutracogenomics, which involves the application of genomic information for improved outcomes in response to disease and disorder. The integration of the study of the interaction among genetic and cellular processes and the dietary environment is called nutrigenomics (Kaput and Rodriguez 2004). The idea of “personalized” or “customized” food and medicine is used in food and nutrition to maintain optimal health and to slow down the development of disease (Palai et al. 2025) (Figure 1).

### 3.2 Phytochemicals

**FIGURE 2:**
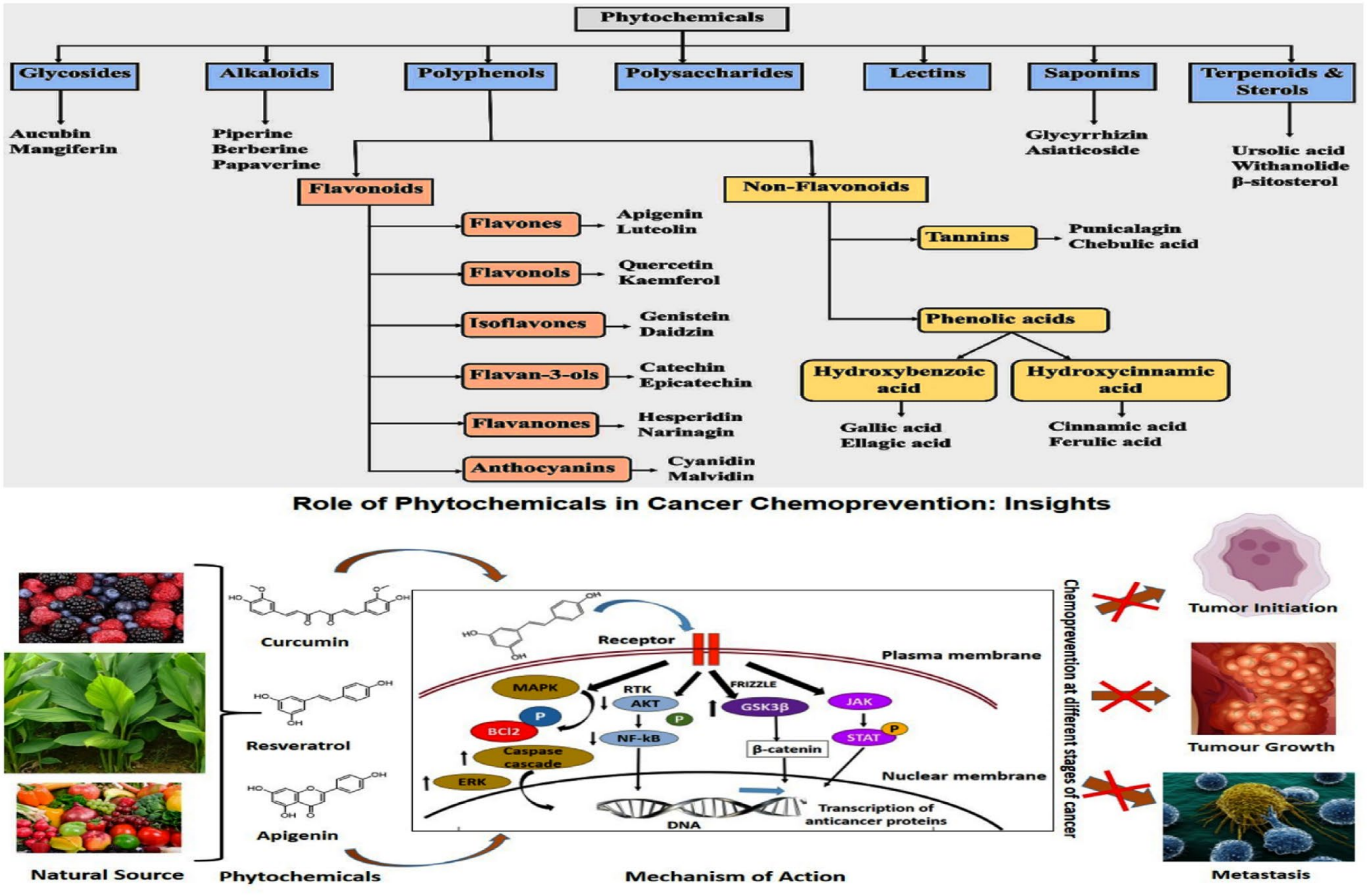
The role of phytochemicals in health sciences. ABTS, 2,2′‐azino‐bis (3‐ethylbenzothiazoline‐6‐sulfonic acid); BMD, bone mineral density; CCL4, carbon tetrachloride; CNS, central nervous system; CVDs, cardiovascular diseases; DNA, deoxyribonucleic acid; EGCG, epigallocatechin‐3‐gallate; EMBASE, Excerpta Medica database; HDL, high‐density lipoprotein; IL‐6, interleukin; LDL, low‐density lipoprotein; MDPI, multidisciplinary digital publishing institute; NO, nitric oxide; PREDIMED, prevención con dietamediterranea; PRISMA, preferred reporting items for systematic reviews and meta‐analyses; PubMed, national library of medicines; RAW264.7 cells, reticulum and white blood cell; RoB 2.0, revised cochrane risk‐of‐bias tool for randomized trials; SAR, structure–activity relationship; SciELO, scientific electronic library online; TNF‐α, tumor necrosis factor; UVB‐induced, ultraviolet B.

For functional foods, there is some science and ethics to support the presence of some phytochemicals and health claims, and the associated health benefits. In the ethnomedical treatment of a variety of ailments, they are components of plants with specific pharmacological and/or physiological effects. They also prevent carcinogenesis that occurs due to UVB, are immunomodulators, neurochemicals, CNS stimulants, analgesics, and diuretics (Gupta and Prakash 2015). Capsaicin is a spicy ginger and red pepper which is anti‐mutagenic and anti‐carcinogenic. Polyphenols (curcuminoids) prevent cancer and cut down inflammation in humans. The genistein group of mice fed with a dietary phytochemical concentrate of soy and the group fed with a dietary soy protein isolate had a 37%–48% smaller malignancy size than the control group. A type of flavonoid, genistein (5,7,4′‐trihydroxyisoflavone), is one of the two main flavonoids found in soy. When it comes to human breast cancer cells, genistein prevents mitogen‐stimulated growth. Carcinogen‐induced breast cancer in rats serves as a model of human breast cancer, and shows that cancer‐initiating and carcinogenic soy isoflavonoid conjugates are chemopreventive (Prakash et al. 2007). Many risk factors are associated with osteoporosis such as hormonal deficiencies, aging and diet. Based on several studies, as shown in Figure 2, phytoestrogens could be used to help postmenopausal women maintain their bone mineral density (BMD) and prevent osteoporosis and other osteoporosis‐related conditions.

There is evidence from a number of human studies that some of the dietary phytoestrogens can have estrogenic effects on women after menopause, including effects on vaginal cytology and reducing the occurrence of hot flashes. Postmenopausal women in both the US and Europe rank cardiovascular diseases (CVDs) as some of the leading causes of their death. ISOs can help decrease the risk of CVDs by using isoflavonoids, soy, soy protein and flax to lower total and LDL cholesterol and raise HDL cholesterol, respectively. The findings from the data corroborate the hypothesis for the reduction of incidence of CVDs in Asian countries due to intake of phytoestrogens (Saklani et al. 2025). Many epidemiological research studies have noted that diets rich in isoflavonoids (phytoestrogens) are associated with a decreased risk of colon, prostate and breast cancer. High plasma enterolactone levels are associated with reduced risk of breast cancer in humans. High levels of enterolactone in the blood are associated with lower risk of breast cancer in humans. Similarly, consumption of lignans and isoflavonoids has been linked with thyroid, ovarian and breast cancer in premenopausal and postmenopausal women. The excessive use of phytoestrogens is the reason why hormone‐dependent cancers are rare in Asia and Eastern Europe. Phytoestrogen consumption negatively correlates with cancer death for breast, ovarian, prostate and colon cancer. There are epidemiological, animal and cell‐line studies that suggest phytoestrogens may be protective against the development of breast and prostate cancer. Eating more tomatoes, dried fruits, lentils, peas, and beans is correlated with a significantly lower risk of prostate cancer (Morabito et al. 2002). Diets rich in phytonutrients are rich sources of phytoestrogens, such as lignans, resveratrol and isoflavones, and exhibit various pharmacological properties. Considering the fact that postmenopausal women are at a higher risk of developing breast cancer, phytoestrogens could be strongly recommended (Cherdshewasart et al. 2009). SAR analysis can be used to identify specific chemical groups in an organism that can have a specific biological effect. Thus, the SAR approach assumes that the structure of a molecule will include the property that gives the molecule its physical, chemical and biological characteristics that make it useful in a therapeutic context in nutraceutical applications.

## 4 Polyphenols: Properties and Pharmacological Applications

**FIGURE 3:**
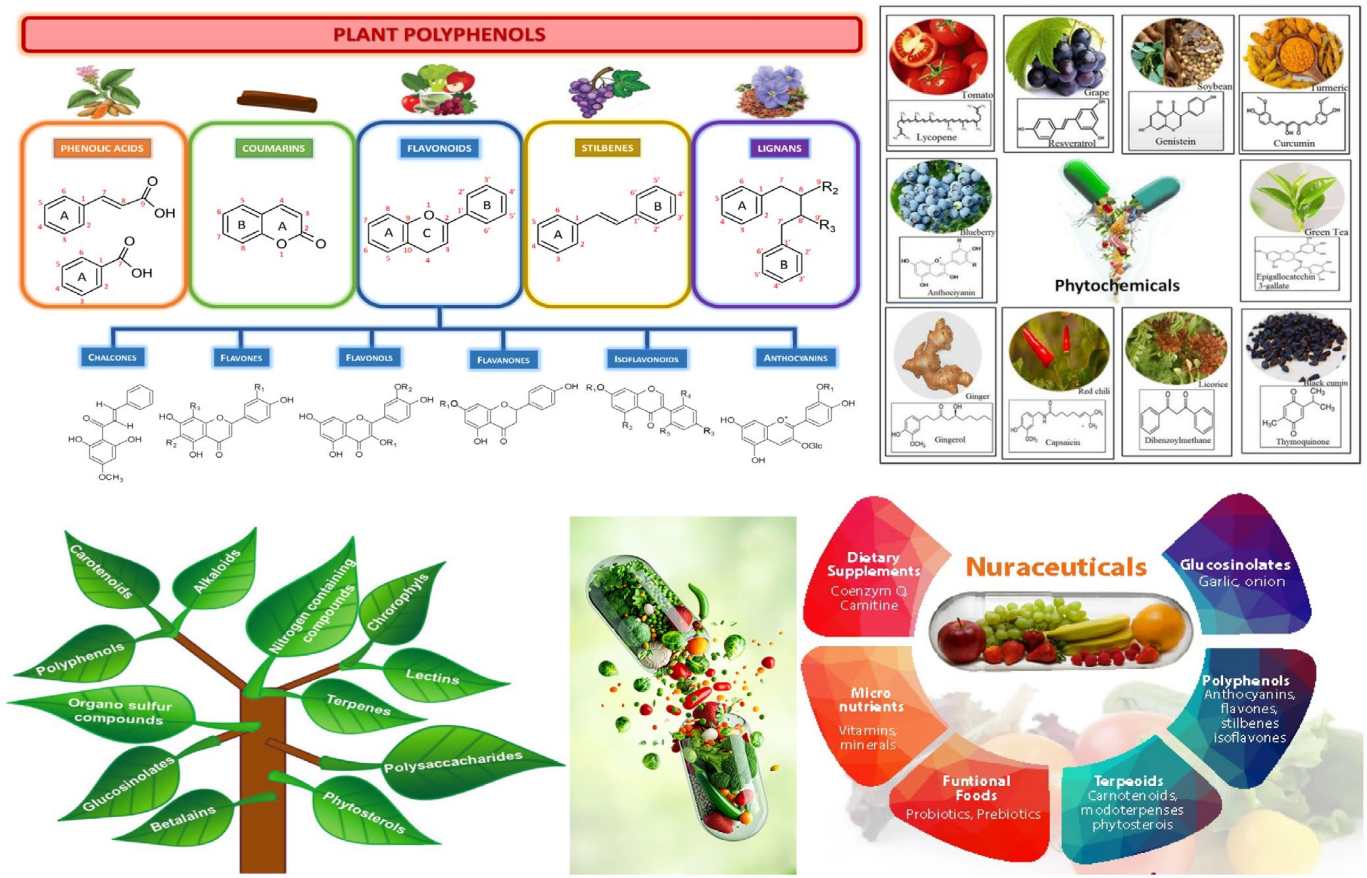
The role of polyphenols and phytochemicals in nutraceuticals and pharmafoods.

Generally, substances with two or more phenolic hydroxyl groups and a benzene ring structure are known as polyphenols. The compounds are classified into two groups: phenolic acids and flavonoids, according to their structure. Flavonoids are found in plant cell vesicles, usually in the form of glycosides. The basic structure of flavonoids is C6C3C6 (3 cyclic units). The flavonoids can be also classified as flavones, isoflavones, flavonols, flavanones, anthocyanins, and chalcones according to their different chemical structures. Flavonoids are required by most plants to develop, grow, flower, fruit, antimicrobial and disease resistance. Various flavonoids have physiological effects that may be beneficial to the human body, such as anti‐cancer, anti‐inflammatory, antibacterial, antifungal, and anti‐oxidant. Polyphenols are found in many fruits, vegetables and beverages. Small‐molecule phenolic compounds have been shown to be water‐soluble in the human digestive system and during processing, and water‐insoluble in the presence of quinic acid and glucose (Bennourab et al. 2020; Rashmi and Negi 2020). The phenolic acids can be divided into three groups depending on the number of hydroxyl groups in the acid as shown in Figure 3, namely, monohydroxybenzoic acid, dihydroxybenzoic acid and trihydroxybenzoic acid.

## 5 Polyphenols Pharmacological Applications

The possibility of utilizing plant polyphenols in the development of functional foods and some of the potential health benefits of these molecules are discussed. The prevention of numerous chronic diseases, such as diabetes, high blood pressure, and cancer, may be greatly aided by functional foods that contain polyphenols. All the pharmacologic benefits of polyphenols are summarized in Table 1.

**TABLE 1:** Different pharmacological applications of polyphenols.

Pharmacological applications	Polyphenols compounds	Assay/methods	References
Anti‐diabetic activity	Catechins, quercetin, epigallocatechin gallate (EGCG), chlorogenic acid, kaempferol, epicatechin, caffeic acid, oleuropein, rutin, curcumin, apigenin, ferulic acid, berberine, and resveratrol	In vitro (α‐amylase, α‐glucosidase inhibition) In vivo and clinical (HbA1c, OGTT)	Nie and Cooper (2021)
Anti‐obesity activity	Epigallocatechin‐3‐gallate (EGCG), genistein, ellagic acid, anthocyanins, gallic acid, curcumin, quercetin, resveratrol, and artepillin C	In vitro (3T3‐L1 adipocytes), In vivo and clinical (BMI and lipid profile studies)	He et al. (2024)
Anti‐cancer activity	Punicalagin, apigenin, luteolin, hesperidin, naringenin, genistein, ellagic acid, resveratrol, caffeic acid, anthocyanins, epigallocatechin gallate (EGCG), curcumin, quercetin, kaempferol, gallic acid, ferulic acid, secoisolariciresinol, cyanidin, and delphinidin	In vitro (MTT assay, Flow cytometry), In vivo and clinical (Western blot, protein expression and RT‐PCR)	Maheshwari and Sharma (2023)
Anti‐viral activity	Delphinidin, cyanidin, quercetin, kaempferol, apigenin, luteolin, gallic acid, caffeic acid, chlorogenic acid, resveratrol, podophyllotoxin, curcumin, coumarins, and phlorotannins	In vitro (Cytopathic effect, MTT and Neutralization assay), In vivo (RT‐PCR and EC_50_, CC_50_, and Flow Cytometry)	Burkard et al. (2025)
Anti‐atherosclerotic activity	Apigenin, cyanidin, ferulic acid, delphinidin, theaflavins, chlorogenic acid, luteolin, naringenin, secoisolariciresinol, resveratrol, curcumin, oleuropein, hydroxytyrosol, epigallocatechin gallate (EGCG), kaempferol, and quercetin	In vitro (LDL Oxidation, MTT and Nitric oxide assay), In vivo (Histopathological analysis), Oxidized LDL (ox‐LDL) and Lipid profile	Palumbo et al. (2025)
Anti‐inflammatory activity	Baicalin, oleocanthal, cyanidin, delphinidin, kaempferol, secoisolariciresinol, apigenin, quercetin, luteolin, epigallocatechin gallate (EGCG), gallic acid, ferulic acid, caffeic acid, and resveratrol	In vitro (COX‐2, LOX and iNOS assay), In vivo (Histopathological analysis), Cell‐based cytokine and ROS studies	Sun et al. (2024)
Anti‐bacterial activity	Apigenin, quercetin, ferulic acid, luteolin, kaempferol, gallic acid, caffeic acid, podophyllotoxin, rhapontin, pinosylvinellagic acid, tannic acid, rutin, rhapontin, and eugenol	In vitro (MIC, agar diffusion and DNA gyrase inhibition), In vivo, and HPLC/LC–MS	Chen et al. (2024)

### 5.1 Anti‐Oxidant Effect

The polyphenols in plants often possess specialities that make them potential anti‐oxidants. Polyphenols are crucial to the human body, and are found in fruits, vegetables, and certain cereals. Polyphenols are antioxidant compounds that have the ability to neutralize free radicals and prevent oxidative damage to DNA (Jakubczyk et al. 2020). In this study, 308 subjects were studied, and 40% of them were men, 27 ± 3.0 years old were the average ages of the respondents and during the supplementation period they consumed 645.0 mg of curcumin per 24 h. Curcumin was shown to have anti‐oxidant activity and malondialdehyde reducing activity (Grzesik et al. 2018) compared to ascorbic acid, glutathione and catechins as anti‐oxidants. All of the maximum reduction equivalents, best ABTS radical scavenging capacity, and capacity to effectively prevent dihydrorhodamine oxidation were all achieved by catechins, in the case of trivalent iron ions. Catechins and other polyphenols have strong anti‐oxidant properties, which are especially valuable for the application of anti‐oxidant therapy and prevention.

### 5.2 Anti‐Inflammatory Effect

Polyphenols from plants can inhibit or kill some inflammatory cells through interfering with the secretion process of these inflammatory cells, or by interfering with cytokines and its receptors. It has been reported in several studies that rutin based hydrogels have the same effect as the conventional drugs in reducing inflammation (Soni et al. 2013). The anti‐inflammatory properties of hesperidin were verified in vitro and in vivo by its ability to inhibit nitric oxide (NO), interleukin (IL‐6) and tumor necrosis factor (TNF‐α) production in RAW264.7 cells in a model of acute lung injury induced by CCL4.

### 5.3 Anti‐Cancer Effect

Polyphenols can be used to prevent some cancerous conditions. They are able to induce apoptosis by toxicizing the cells and inhibiting the growth of tumors. Resveratrol possesses strong anti‐oxidant activity, an apoptotic effect and cell growth inhibition, which could affect the development of cancers and other related diseases (Lee and Lee 2021). In terms of their findings, quercetin has been greatly applicable in the treatment and prevention of esophageal cancer. Various investigations, which are associated with polyphenols, have anti‐cancer properties. Epigallocatechin gallate (EGCG) and curcumin showed good anti‐cancer property in breast cancer, but the other compounds such as silymarin have been reported to induce apoptosis in liver cancer cells and have good prevention and therapeutic potential in liver diseases (Chandra, Gahlot, et al. 2023).

### 5.4 Anti‐Microbial Effect

Polyphenols are effective in inhibiting a wide variety of microorganisms. This is particularly for flavonoids with a greater antibacterial activity than other polyphenols. According to the majority of studies, polyphenols have good antibacterial properties, and can be combined with antibiotics. The antibacterial activity of curcumin against Rhizopus solani and *Staphylococcus aureus* was high in chitosan films incorporating curcumin (Liu et al. 2016). Furthermore, it has been well reported and confirmed that the tea polyphenols, silymarin and rutin have antibacterial properties (Yang and Cheng 2019).

### 5.5 Pro‐Oxidant Effect

Polyphenols can cause damage to DNA at high concentrations and trigger apoptosis (Gunes‐Bayir et al. 2017). The pro‐oxidant properties accelerate the aging process and the pro‐oxidant activity shortens chronological life span of brewer's yeast (Canedo‐Santos et al. 2022). Owing to its lipid oxidation, protein carbon formation, and thiamine group oxidative losses preventing characteristics, gallic acid affects the structural characteristics and the natural reactions of proteins (Cao et al. 2016). The meat pigs fed high levels of soybeans had more severe oxidative changes in the liver, plasma and fat. They were also very pro‐oxidant and enhanced their overall anti‐oxidant capacity and superoxide dismutase activity by 64 days (Chen et al. 2016).

### 5.6 Anti‐Diabetic Effect

Consuming a diet that is rich in polyphenol may help to lower the risk of diabetes. Several studies have proven that polyphenols control the insulin cascade and increase insulin sensitivity in peripheral tissues (Avila et al. 2017). α‐glucosidase and α‐amylase, which control blood sugar and regulate its absorption in the intestines, are greatly inhibited by multiple polyphenols (Vayalil 2012). Polyphenols, such as catechins, that are effective anti‐oxidants and anti‐diabetics are abundant in many foods, such as asparagus. A comprehensive and systematic evaluation and characterization of the molecular mechanisms of action, modes of action, and effects of quercetin both in vitro and in vivo have been shown to have excellent preventative and therapeutic potential for diabetes (Eid and Haddad 2017). Therefore, polyphenols are potential for prevention and treatment of diabetes.

### 5.7 Anti‐Hypertensive Effect

Several polyphenols, including cocoa, which has high levels of flavanol compounds such as proanthocyanidins and catechins, may induce vasodilation, improve endothelial activity and reduce the oxidative susceptibility of LDL. This was pointed out by the European Food Safety Authority (Williamson 2017). The study (Huang et al. 2013) provided an extensive review of the positive effects of phenolic acids, tannins, resveratrol, anthocyanins, flavonoids, flavanols and other polyphenols on the activity of vasodilators and other activities that affect blood pressure. The vasodilatory effect of curcumin alone, amlodipine alone and curcumin in combination with amlodipine has been tested in separate studies in isolated rat aortic rings. The researchers concluded that, when people who were on antihypertensive drugs took turmeric or curcumin, either as a dietary supplement or for medical reasons, their antihypertensive effects were not altered (Lee et al. 2021). This provides a good basis to use curcumin and other antihypertensive polyphenols as food additives to prevent and treat hypertension.

### 5.8 Anti‐Obesity Effect

Polyphenols can impact obesity in several ways, including by increasing the breakdown of fat, the burning of fat, the killing of fat cells, and decreasing the number of fat cells. Green tea, the drink that has numerous health benefits and has potential to help treat obesity, is packed with gallocatechin gallate, catechin, and other polyphenols including catechins (Sung et al. 2010). The anti‐obesity effect of soybean has been attributed to the activation of hormone‐sensitive lipase (HSL) for the activation of lipolysis, and suppression of pancreatic lipase (PL) and pancreatic protein lipase (PPL) for the inhibition of adipocyte proliferation. Similarly, quercetin was seen to inhibit the growth of adipose tissue in obese rats in one of the studies (Ting et al. 2018) and was therefore said to have anti‐obesity properties.

### 5.9 Anti‐Atherosclerotic Effect

Polyphenols are compounds with beneficial properties to mitigate and treat atherosclerosis and have been sought after for functional food development and application. Others, plant polyphenols could help the coronary arteries by strengthening the HDL, decreasing the LDL, and preventing its oxidation (Bahramsoltani et al. 2019). Resveratrol and ellagic acid can enhance the activity of the endothelial barrier that can anti‐atherosclerotic act (Qiu et al. 2018). Moreover, EGCG has been discovered to have some anti‐atherosclerotic properties; the report provides a detailed description and diagram of this effect (Tanaka et al. 2012).

## 6 Bio‐Based Polymer Nano‐Delivery of Polyphenols

Polyphenols can be better delivered using biopolymers due to several important properties of the latter, such as biodegradability and biocompatibility. Different studies have been conducted to study the design, production, transportation, encapsulation and preservation of polyphenols with nanocarriers based on proteins, polysaccharides and lipids (Zhang et al. 2021; Rambaran 2022). The development of functionalized goods with polyphenols is an interesting innovation (Table 2) and it certainly provides a reference for the development of new nutrition and health products with polyphenols. Wacker Chemie AG has developed increased absorption properties of Cavacurcumin by combining it with γ‐cyclodextrin (Hundshammer et al. 2021; Lozano‐Perez et al. 2017) and has studied the encapsulation, adsorption and release of quercetin using silk protein nanoparticles. In intestinal fluid simulations, encapsulated quercetin kept its potent free radical scavenging capabilities and showed a slow‐release effect. Likewise, curcumin and other polyphenols can be encapsulated, released and delivered over time in the gastrointestinal tract using whey protein nanoparticles. However, the system is very theoretical and needs to be stable at pH 7 for use in functional beverages (Solghi et al. 2020). The incorporation of proteins as protein building‐blocks has been widely employed to prepare nanocarriers to entrap polyphenols and enhance their bioactivity (Marziyeh et al. 2018; Ba et al. 2020).

**TABLE 2:** An overview of the nanotoxicity of polyphenol‐loaded nanomaterials.

Disorder/disease	Nanocarrier	Loading	Polyphenols	Cell lines	Results	References
Cancer	PLGA	Conjugation	Curcumin	KB V1 and KB 3‐1 cells	Cur‐NPs more binding as compared t KB V1 and KB 3‐1 cells. Comparison of KB 3‐1 cells, Cellular absorption of Cur‐NPs APgp is graeter than KB V1 cells	Punfa et al. (2012)
	Schizophyllan and chitin nanoparticles	Encapsulation	Ellagic acid	MCF‐7 cells	EA‐SPG‐NP and EA/Ch‐NP effectively decreased the proliferation of breast cancer cell lines, IC50 value, 60 and 115 g/mL	Pirzadeh‐Naeeni et al. (2020)
	Nanocapsules	Encapsulation	Curcumin and quercetin	MCF‐7 cells	Cytotoxicity of bioactive compounds greater than free nature	Ghayour et al. (2019)
Neurological	CeO2@SiO2 PEG nanoparticles (CSP‐ NPs)	Encapsulation	Proanthocyanidins and curcumin	PC‐12 cells	CSP NPs significant reach proanthocyadinins and curcumin to inhibit strong neuroprotective impact against A1‐42 mediated toxicity and recovered cell viability, 57.5%–81.0% at 25.0 g/mL	Chen et al. (2020)
	PLGA‐NPs	Encapsulation	4‐hydroxyisophthalic acid	PC‐12 cells	4 HIA and 4 HIA encapsulation PLGA, reduced hydrogen peroxide (H_2_O_2_) induced toxicity with 84%–94% cell viability	Ravikiran et al. (2021)
Cardiovascular	Pluronic F‐127	Encapsulaion	Curcumin and resveratrol	H9c2 cells	Combination of curcumin and resveratrol as free drugs or combined, inhibited apoptosis and sequestered (ROS in H9C2 Cells)	Carlson et al. (2014)

Furthermore, by making various technical adjustments, polysaccharide‐based nanomaterials can be developed to transport and encapsulate bioactive compounds in various applications such as the preservation of polyphenols, as was done by preparing starch nanoparticles of cassava starch loaded with curcumin (Qin et al. 2020; Geetha and Alummoottil 2015). Being highly inter‐solubility and biodegradable, chitosan has been extensively studied and researched as the carrier of polyphenols as it is a naturally produced polysaccharide. The use of chitosan nanoparticles can be used effectively to release polyphenols, such as polyphenols from tea, in a controlled manner with a higher bioavailability (Liang et al. 2017).

Polyphenols are usually core‐encapsulated in lipid nanocarriers as they are biocompatible and biodegradable, similar to proteins and polysaccharides. Bioactive systems of solid lipid nanoparticles can be used to effectively deliver hydrophobic nutraceuticals. Using solid lipid nanoparticles, for instance (Li et al. 2021), transport and encapsulate resveratrol. The researchers determined that these resveratrol nanostructures caused an anti‐fatigue effect in the high‐intensity exercise group, and improved the anti‐oxidant protection, which may be beneficial in their application as a delivery system. Similarly, additional polyphenols, such as quercetin, had their bioavailability greatly enhanced by solid lipid nanocarriers (Talarico et al. 2021).

Though all this, there remain many challenges to be cleared before the nanoformulated phytochemical can be put to use in therapeutic applications. One of the primary problems is the regulatory uncertainty. Global regulatory bodies, such as the European Medicines Agency (EMA), the Food and Drug Administration (FDA) in the United States, and other national authorities, have not yet developed completely standardized rules that particularly address nanonutraceuticals and phytochemical‐based nanomedicines.

Besides, liposomes and nanoemulsions are well supported in enhancing the bioavailability of plant polyphenols, and are effective delivery vehicles of lipophilic polyphenols. The research work conducted by Lital was in‐depth and focused on the transport, encapsulation and protection of lipophilic functional components such as polyphenols, tastes and pigments, involving the use of nanoemulsions. The kinetic, metabolic and biological effects of hydrophobic substances can be enhanced by supplying polyphenols using nanoemulsions and making them more soluble. This will serve as a significant point of reference for the application of plant polyphenols as healthy concoctions in food sector using nanoemulsion. The use of liposomes for delivering various functional substances in food systems was also studied recently and it was found that the small size, biodegradability, non‐toxicity, and unique amphiphilic nature of liposomes make them excellent delivery sources (Emami et al. 2016).

Moreover, nanoparticles comprising polysaccharide‐coated protein can enhance the stability of delivery systems. For example, zein can be mixed with other polysaccharide protein composite nanoparticles including pectin, carrageenan and chitosan. This may improve the transport capacity of composite particle and its protecting effect to polyphenols, which is the shortcoming of the tendency for single zein nanoparticles to aggregate. To obtain zein‐rhamnolipid composite nanoparticles (NPs), Dai and co‐workers blended zein and rhamnolipids (RLs). The study has revealed that the composite nanoparticles could successfully seal and secure curcumin and can be employed as an alternative nanocarrier in the functional foods and beverages to transport hydrophobic medical products (Nagime et al. 2025).

Numerous polyphenol nanoparticles have been developed to enhance bioavailability and stability, including micelles, nanosomes, liposomes, emulsions, lipid and phospholipid nanoparticles. Epigallocatechin‐3‐gallate is the most popular and used polyphenol, while resveratrol and curcumin are poorly absorbed. All developed nanoparticles may have the ability to penetrate the blood–brain barrier (BBB), which plays a vital role in neuroprotection and half‐life (Dayar and Pechanova 2021). Thus, anti‐P‐glycoprotein loaded curcumin loaded PLGA nanoparticles showed lethal effect in human cervical cancer KB‐3‐1 and KB‐V1 cells, besides decreasing the cell viability, and increasing the solubility and uptake of curcumin in cells. The ellagic acid complexes with schizophylan and chitin nanoparticles and ferric‐coordinated polyphenols nanoparticles were used to induce PTT‐assisted ferrous therapy in MCF‐7 breast cancer cells to exploit their anti‐cancer properties. A cell viability experiment revealed significant antiproliferative effects when compared to the MCF‐7 cells, with increased effects at high doses (Anand et al. 2022) (Table 2).

Numerous compositions of nanoparticles work very well in the lab but have problems in the production process. Another important factor that is important with the nano‐delivery systems is the assessment of safety and toxicity. The toxicity profile can vary depending on the composition of the particles, their size, surface charge, shape, dosage, and administration. So standard in vitro and in vivo toxicity tests are essential prior to clinical use. Many polyphenol and phytoconstituent nanoformulations have been demonstrated with enhanced pharmacokinetic and pharmacodynamic properties in animal models. Improved scalable manufacturing techniques, clinical validation based on evidence, development of rigorous clinical safety assessment procedures, and harmonization of regulatory frameworks should be the priorities of future research. Combination of nanotechnology and precision medicine, nutrigenomics, AI, and targeted delivery could enhance polyphenol and phytoconstituents' therapeutic value even further.

## 7 Toxicity and Safety

Certain foods and beverages possess polyphenols, which may help people's health, particularly in managing and preventing age‐related diseases and disorders like type‐2 diabetes and obesity. The results of “in vivo” studies can be used for assessment of the acute, subacute, chronic and even potential long term oral toxicity of substances. They are the negative impact of environmental pollutants on reproductive system. The impact of Cistanches herba on male mice's reproductive systems was investigated in an experiment. The sperm count of the mice treated with C. herba reduced dose dependently with significant reduction at the doses 250, 500 and 1000 mg/kg of the dose. The *C. tubulosa* extract, Memoregain, showed favorable safety profiles with no genotoxic effects in a range of tests including the Ames test and the mammalian micronucleus test and the chromosomal aberration test. In addition, curcumin has a significant antioxidant effect, and a clinical trial with an average age of 27 ± 3.0 years and an average dose of 645 mg/24 h showed that curcumin lowers the levels of malondialdehyde. Polyphenols can stop many diseases by removing free radicals and hindering DNA oxidation. Low to medium dose of green tea (0.01%–0.1% diet) protects against liver damage in mice. The European Food Safety Authority (EFSA) has approved anthocyanins as a food additive in the EU. A low dose of quercetin (1500–5000 μmol/L) was found to stimulate cell growth while high doses inhibited the growth of cancer cells. Following the polyphenols or 50 mg/day of isoflavones or 100–300 mg/day of grape seed extracts high in proanthocyanidins are obtained by using soy products in Japan or grapes or wine in some European nations (Zhang et al. 2022).

At the recommended therapeutic and/or dietary doses, polyphenols and other phytochemicals demonstrate a relatively low toxicity profile and seem to be safe. These are widely used in dietary supplements, medicinal plants and as nutraceuticals, supporting their overall safety and efficacy in supporting health. But adverse effects like gastrointestinal upset, liver or kidney toxicity, interference with absorption of certain nutrients, and changes in the metabolism of certain drugs may occur when these substances are taken in excess. Extensive toxicological studies, quality control and well‐designed clinical trials are essential for establishing safe dosages and safety over time. The available data suggests that polyphenols and phytoconstituents have a favorable safety profile and can be considered safe for the development of nutraceuticals, therapeutic and preventive health agents.

## 8 Discussion

Polyphenols can have many possible applications as functional food additives. With the ongoing advances in using nanotechnology to deliver bioactive compounds, there has been a growing number of studies and development of food grade nanocarriers. This remains a barrier to the development of nutritious meals. Although medicinal plants have been used by indigenous people to treat various problems since time immemorial, the physiological and therapeutic efficacy of phytonutrients and phytochemicals in the prevention of treatment of most diseases has not been scientifically proven until recently. Plant‐based nutraceuticals that are “novel” can be very useful in disease‐preventive diets. The advantages of polyphenols are due to their excellent properties; their high nutritional value, low toxicity, biocompatibility and biodegradability mean that they can be encapsulated and distributed in protein form. They can be mixed or modified and are available in various forms including hydrogel fibers, thin films and nanoparticles, and enhance their surface activity and increase their bioavailability (Ba et al. 2020). These encapsulating methods can be used to enhance the water dispersibility of polyphenols, gastrointestinal stability, environmental stability, tartaric acid, and extended release. For example, hydrophobicity, electrostatic interaction and hydrogen bonding of curcumin and zinc ions are the key parameters that play a role in the nanoencapsulation (Kasaai 2018). The racurcumin also has exhibited potential pharmacokinetic properties in humans clinical trials (Naksuriya et al. 2014).

The physicochemical properties have been improved to a great extent by using nanotechnology in the preparation of the polyphenol nanocomposites. In addition, the geometry, surface, drug carrying and targeting properties of the compounds are unique. Biological nanocomposites supported with polyphenols are used in various applications such as cosmetics, functional foods, health care products, etc. The physical and chemical characteristic of the carriers depends on the packaging material. According to some studies, the use of protein and chitosan as ingredients for preparation of carrier formulation is more common. In many studies, they have also been proven to have positive impacts on intestinal absorption and bioavailability of plant polyphenols (Guan et al. 2022). Nutraceutical clinical trials are supposed to determine the effectiveness and safety of such products, which are typically sourced from food, and can have physiological benefits as mentioned in Table 1.

## 9 Conclusion

Plant polyphenols have been recognized and used as food ingredients in sustainable, healthy and functional foods loaded and carried by nanocarriers.

They possess a broad spectrum of biological activities such as anti‐diabetic (Tomar et al. 2024), antioxidants, anti‐inflammatory, antibacterial (Bisht et al. 2024), anticancer, cardioprotective, anti‐viral (Chandra, Palai, et al. 2023) and neuroprotective properties, and are used as ingredients in functional foods and nutraceuticals. Advances in the field of nutrigenomics have also shown the potential of these phytochemicals to affect gene expression, cellular communication pathways, and metabolism, paving the way for personalized nutrition and precision medicine. While there are problems with bioavailability and stability, and issues of clinical validation, new technologies are being developed that offer exciting possibilities for enhancing their therapeutic potential, such as nano‐formulations and advanced delivery systems. Interdisciplinary research that combines the fields of phytochemistry, nutraceutical science, nutrigenomics, and clinical studies will be required to facilitate the translation of these health‐promoting polyphenols and other phytoconstituents into clinical practice for the prevention and management of disease.

Establishing molecular mechanisms of action, enhancing bioavailability through novel delivery methods, developing standardized quality‐control procedures, carrying out thorough clinical trials, and incorporating nutrigenomics techniques for tailored nutrition and treatment should be the main goals of future research. In the process of doing this, polyphenols and phytoconstituents will be successfully moved from the bench to clinically proven pharmaceutical, nutraceutical, and functional food products, which will enhance human health and prevent disease around the world.

## Author Contributions

**Subhash Chandra:** conceptualization, writing – original draft, writing – review and editing, methodology, validation, investigation, formal analysis, project administration, software, data curation, supervision, resources, visualization. **Atasi Goswami:** conceptualization, writing – original draft, writing – review and editing, validation, methodology, software. **Md. Sakib Al Hasan:** conceptualization, writing – original draft, writing – review and editing, formal analysis. **Md. A. K. Azad:** review and editing, data curation, software formal analysis.

## Funding

The authors have nothing to report.

## Conflicts of Interest

The authors declare no conflicts of interest.

## Data Availability

The authors have nothing to report.
